# WGGLFA: Wavelet-Guided Global–Local Feature Aggregation Network for Facial Expression Recognition

**DOI:** 10.3390/biomimetics10080495

**Published:** 2025-07-27

**Authors:** Kaile Dong, Xi Li, Cong Zhang, Zhenhua Xiao, Runpu Nie

**Affiliations:** 1 School of Electrical and Information Engineering, Wuhan Institute of Technology, Wuhan 430205, China; 22303010002@stu.wit.edu.cn (K.D.); 22303010041@stu.wit.edu.cn (R.N.); 2 College of Information and Artificial Intelligence, Nanchang Institute of Science and Technology, Nanchang 330108, China; mattzhang@wit.edu.cn

**Keywords:** facial expression recognition, deep learning, multi-scale feature, global–local feature fusion

## Abstract

Facial expression plays an important role in human–computer interaction and affective computing. However, existing expression recognition methods cannot effectively capture multi-scale structural details contained in facial expressions, leading to a decline in recognition accuracy. Inspired by the multi-scale processing mechanism of the biological visual system, this paper proposes a wavelet-guided global–local feature aggregation network (WGGLFA) for facial expression recognition (FER). Our WGGLFA network consists of three main modules: the scale-aware expansion (SAE) module, which combines dilated convolution and wavelet transform to capture multi-scale contextual features; the structured local feature aggregation (SLFA) module based on facial keypoints to extract structured local features; and the expression-guided region refinement (ExGR) module, which enhances features from high-response expression areas to improve the collaborative modeling between local details and key expression regions. All three modules utilize the spatial frequency locality of the wavelet transform to achieve high-/low-frequency feature separation, thereby enhancing fine-grained expression representation under frequency domain guidance. Experimental results show that our WGGLFA achieves accuracies of 90.32%, 91.24%, and 71.90% on the RAF-DB, FERPlus, and FED-RO datasets, respectively, demonstrating that our WGGLFA is effective and has more capability of robustness and generalization than state-of-the-art (SOTA) expression recognition methods.

## 1. Introduction

Facial expression conveys extensive cues about emotional states and interpersonal attitudes during human communication. They not only effectively communicate an individual’s internal emotions but also provide a way for others to perceive and understand these emotions. Facial expression recognition (FER) has been widely applied in affective computing [1,2], medical diagnostics [3], and various other fields. In recent years, multimodal emotion recognition methods such as the integration of facial, speech, and textual information have become an important research direction in affective computing and have achieved notable progress [4,5,6]. However, facial expressions, as the most direct and intuitive emotional cues, still face challenges in real-world scenarios, including occlusion, illumination variation, and pose changes. Therefore, research focusing on single-modality facial expression recognition remains of significant theoretical and practical importance. In the early stages, FER demonstrated superior recognition performance on standard laboratory datasets [7,8,9] under strictly controlled conditions. These datasets primarily consist of facial emotions deliberately expressed by subjects under controlled conditions, where faces are oriented forward with no obstructions, resulting in high recognition accuracy [7,10]. Examples of such datasets include CK+ [11] and MMI [12], which feature unobstructed facial expressions. However, when the recognition context transitions from controlled laboratory settings to dynamic real-world environments, the performance of facial expression recognition (FER) systems deteriorates significantly. Experiments have shown that recognition accuracy on unconstrained datasets [13,14] is considerably lower compared to controlled laboratory datasets [15,16,17]. To alleviate the facial expression feature loss caused by various factors in unconstrained environments, early approaches typically treated the entire face image as a holistic input. Cornejo et al. [18] utilized robust principal component analysis (RPCA) to restore facial regions affected by occlusion or data loss. Yao et al. [19] presented HoloNet, which improved performance by combining residual structure with the CReLu activation function to increase depth and width. Bourel et al. [20] designed an improved Kanade–Lucas tracking algorithm to correct drifting or missing facial keypoints. Zhang et al. [21] integrated geometric features and Gabor wavelet features, with experiments showing significant improvements in recognition accuracy. To address the challenges in real-world facial expression recognition (FER) tasks, recent investigations have increasingly concentrated on local facial feature extraction. Liu et al. [22] proposed the symmetric multi-scale residual network (SMResNet), which used three branches to independently extract characteristics from the mouth, eyes, and entire face. Experimental results demonstrated that the fusion of these three branches significantly outperforms using a single regional branch. Liu et al. [23] proposed the graph regularized discriminative nonnegative matrix factorization (GDNMF) method, which simultaneously modeled local geometric structures and class label information and achieved superior clustering performance across multiple image datasets. Li et al. [16] designed a learning framework (ACNN) that combines a gate unit and integrates full-face and key regions to improve expression recognition performance under occlusion. Although these methods contribute to the reconstruction of occluded features, they suffer from high computational complexity, increasing system cost. Zhao et al. [24] introduced the discrete wavelet transform (DWT) for facial expression feature extraction, effectively reducing computational cost. However, its application remains primarily limited to shallow feature extraction, with insufficient integration of deep semantic modeling and attention mechanisms, thereby hindering the full exploitation of its potential in frequency representation. Indolia et al. [25] proposed a method combining self-attention and discrete wavelet transform (DWT), which enhances local feature extraction in facial expression recognition, but it is still limited in capturing the structural details across multiple scales.

The falcon is among the most visually acute animals in nature, possessing a multi-layered visual system that enables both global perception and precise detection of local details. Its retina contains two foveae, specialized for long-distance focusing and high-resolution near-field perception, respectively. Inspired by this sophisticated visual mechanism, we propose a wavelet-guided global–local feature aggregation network (WGGLFA) for facial expression recognition. Our WGGLFA network consists of three main modules: the scale-aware expansion (SAE) module, the structured local feature aggregation (SLFA) module, and the expression-guided region refinement (ExGR) module. All three modules leverage the time–frequency locality of the wavelet transform to achieve high- and low-frequency separation modeling, thereby enhancing the fine-grained representation of facial expressions under frequency domain guidance. Specifically, in the scale-aware expansion (SAE) module, we integrate dilated convolutions [26], wavelet convolution (WTConv) [27], and a multi-receptive field fusion mechanism. The multi-receptive field approach is implemented through a branching structure of dilated convolutions with varying dilation rates. By assigning different dilation rates to each branch, the network can effectively model information across different spatial scales, thus enabling hierarchical perception from local to global contexts. Furthermore, we employ a wavelet-based decomposition method to capture multi-band features, allowing the network to model low-frequency and high-frequency components separately. The low-frequency features mainly contain object contours and region-level geometric structures, whereas the high-frequency features represent texture, edges, and other fine details. By independently processing and subsequently fusing features from different frequency bands, the network is able to simultaneously focus on both global structures and local details, thereby achieving synergistic multi-scale information representation. In the structured local feature aggregation (SLFA) module, we perform adaptive partitioning of the facial region based on facial keypoints to accurately extract local features. We combine wavelet decomposition with the efficient local attention (ELA) to jointly model structural patterns and edge responses across multiple frequency components, thereby improving the completeness and discriminative capacity of local representations. Given the importance of high-response regions for expression recognition, we guide the network to attend to emotionally salient areas through the ExGR module, enhancing robustness under complex conditions. We summarize the main contributions of this study as follows:

1. We propose a wavelet-guided global–local feature aggregation network (WGGLFA), which integrates multi-scale feature extraction, local region feature aggregation, and key region guidance. We utilize the spatial frequency locality of the wavelet transform to achieve high-/low-frequency feature separation, enhancing fine-grained representation.
2. The scale-aware expansion module is designed to enhance the ability to capture multi-scale details of facial expressions by combining wavelet transform with dilated convolution.
3. The structured local feature aggregation module is introduced, which dynamically partitions the facial regions based on facial keypoints and enhances the partitioned feature units. These representations are then fused with those extracted from high-response regions by the expression-guided region refinement module, improving the accuracy of fine-grained expression feature extraction.
4. Extensive experiments on RAF-DB, FERPlus, and FED-RO demonstrate that our WGGLFA is effective and has more robustness and generalization capability than the SOTA expression recognition methods.

## 2. Related Work

Facial expression recognition techniques are typically divided into handcrafted and deep learning-based methods based on how features are extracted. Handcrafted feature-based recognition methods mainly include HOG [28], SIFT [29], LBP [30], and Gabor wavelet [31]. Zhan et al. [32] integrated the Gabor wavelet transform with elastic template matching for facial expression recognition. Shao et al. [33] combined NSCTLBP and Gabor features for facial expression recognition, which outperformed single-feature approaches under various conditions. Although handcrafted feature extraction methods offer certain advantages in some tasks, they often face issues of high computational complexity and limited adaptive learning capability.

Since Hinton and others proposed Deep Belief Networks in 2006, deep learning technology has attracted widespread attention. These methods use multi-layer neural networks to model high-dimensional data and effectively extract and represent features. Deep learning has increasingly taken the place of handcrafted feature methods in facial expression recognition (FER), becoming a major research direction [34,35,36,37,38,39,40]. At the initial stage, AlexNet [41] and VGGNet [42] used a stacked convolutional module approach, enabling the model to capture multi-level features from global to local scales. GoogLeNet [43] used convolutional kernels of varying scales for feature extraction at multiple levels. The Res2Net block [44] introduced residual connections from ResNet [45], enabling finer changes in the receptive field. Although these methods are optimized for specific challenges, they still suffer from issues such as high computational complexity and complex network structures. Inspired by Res2Net [44], Xia et al. [46] proposed MM-Net, which used grouped residual modules and a channel grouping strategy to aggregate multi-scale features. By incorporating depthwise separable convolutions, this approach reduced computational cost and enhanced expression recognition performance in complex scenarios. Zhao et al. [47] introduced MA-Net, which incorporates multi-scale representations and localized attention mechanisms to enhance feature learning in occluded scenarios. Ali et al. [48] used Radon projection with translation and rotation invariance along with Discrete Wavelet Transform (DWT) for better expression classification through multi-level feature learning. Wang et al. [49] proposed UFSRNet, which adopted Discrete Wavelet Transform (DWT) to retain high-frequency features, thereby effectively restoring facial details while reducing the number of parameters and improving recognition accuracy. Ezati et al. [50] introduced a hierarchical structure that combines dilated and standard convolutions to capture features (LANMSFF). Although this innovation enhanced the performance of multi-view facial expression analysis, it lacks effective modeling of critical local regions. Shahzad et al. [51] proposed ZFER, which enhanced sub-region features. Facial key points were detected using MTCNN [52] and divided into four parts. A pre-trained VGG-16 was used to extract features, which were classified by the network after channel stacking. Tao et al. [53] introduced a hierarchical attention framework that adaptively emphasizes key regions and integrates multi-level features, demonstrating its effectiveness in multi-scale facial expression recognition. Similarly, Liu et al. [54] introduced AMP-Net, which leverages facial feature cues and human face perception mechanisms, combining facial features from global, local, and visually significant facial regions. Wang et al. [17] proposed an attention framework called RAN, which integrated a spatially biased loss to enhance focus on key facial areas. Although the above methods have achieved progress in integrating local and global features, some approaches used static region partitioning [51,52], ignoring individual facial geometry. The method in [54] used complex attention mechanisms, increasing computational cost.

In addition, transformer [55] architectures have attracted increasing attention in facial expression recognition (FER) owing to their capacity for capturing long-distance feature relationships. ViT (vision transformer)-based methods [56] and hierarchical variants such as Swin-FER [57] have achieved competitive performance through modeling global semantic relationships. Xu et al. [58] proposed the global–local feature fusion transformers (GFFT), utilizing self-attention fusion to achieve cross-patch feature interaction. However, models typically involve high computational complexity and rely on large-scale datasets for effective training [55,56].

In recent years, multimodal emotion recognition methods that integrate visual, audio, and textual information have attracted widespread attention. These methods typically improve the robustness of emotion recognition through multimodal alignment and cross-modal attention mechanisms. Wang et al. [59] proposed a multi-granularity cross-modal alignment framework (MGCMA), which enhances the collaborative modeling of emotional information between speech and text through distribution-level, token-level, and instance-level alignment strategies. Pan et al. [60] proposed a hybrid fusion model (MMAN) with a directional attention-based module for early fusion of speech, text, and visual features, thereby improving information interaction efficiency in multimodal emotion recognition. Ryumina et al. [61] proposed the first multi-corpus multimodal emotion recognition method, which employs dedicated encoders for audio, video, and text, and integrates features using a gated attention mechanism, achieving strong performance on the task. However, multimodal methods often rely on synchronized multi-source inputs, which increases data collection costs and complicates cross-modal alignment. Therefore, fine-grained modeling of facial expressions under a single visual modality remains a key component for improving the overall performance of multimodal systems. Our study focuses on detailed facial expression modeling within the visual modality to provide extensible local and global feature representations for future multimodal emotion recognition.

## 3. Methodology

**Figure 1:**
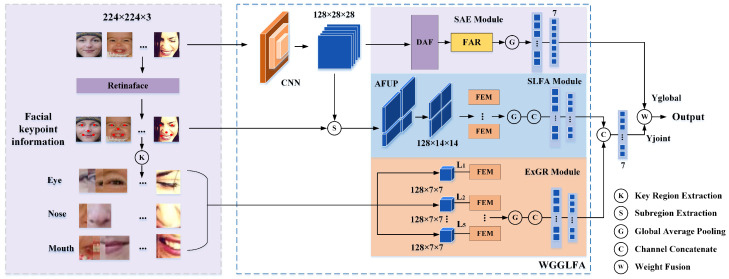
Overall architecture of the proposed wavelet-guided global–local feature aggregation network (WGGLFA). The WGGLFA network is enclosed in the dashed box. A convolutional neural network (CNN) is employed for extracting low-level feature maps. WGGLFA consists of three main modules. The scale-aware expansion (SAE) module includes the dilated fusion (DAF) and feature recalibration (FAR) submodules. The structured local feature aggregation (SLFA) module employs an adaptive feature unit partitioning (AFUP) strategy to divide the feature maps into four subregions based on the locations of facial keypoints. Subregion extraction (denoted by S) refers to the partitioning of the feature map into structured subregions based on the spatial distribution of facial keypoints. The expression-guided region refinement (ExGR) module expands outward from the facial keypoints to generate five fixed regions, which correspond to the left eye, right eye, nose tip, and the left and right corners of the mouth, and is followed by a feature enhancement module (FEM) for further refinement of these local regions. Key region extraction (denoted by K) refers to the generation of semantically important regions (such as the eyes, nose, and mouth) by expanding outward from facial keypoints. The structured local feature $Y_{joint}$ is fused with the global feature vector $Y_{global}$ to produce the final expression representation.

As shown in Figure 1, we propose a wavelet-guided global–local feature aggregation network (WGGLFA) to address the challenge of capturing multi-scale structural details in facial expressions. WGGLFA is composed of three key components: the scale-aware expansion (SAE) module, the structured local feature aggregation (SLFA) module, and the expression-guided region refinement (ExGR) module. All three modules utilize the spatial-frequency locality of the wavelet transform to achieve high-/low-frequency feature separation, thereby enhancing fine-grained expression representation under frequency domain guidance.

We employ the RetinaFace [62] to localize five representative facial keypoints, including the centers of the eyes, the tip of the nose, and the corners of the mouth, to support subsequent analysis. These keypoints provide structural guidance for the following stages. Input images are resized to 224×224×3. A convolutional neural network (CNN) is employed to extract low-level features, resulting in a feature map with a size of 128×28×28. The SAE module is composed of the dilated fusion (DAF) module and the feature recalibration (FAR) module. The SAE module integrates wavelet transform with dilated convolution to capture the multi-scale structural details present in facial expressions. We first feed the low-level features into the DAF module. In this stage, wavelet transform is used to separate high- and low-frequency components by leveraging its spatial-frequency localization. This helps preserve important low-frequency structural information. We then apply multi-scale dilated convolutions to model large-scale facial features such as the contours of a smile or the structure of the eyebrows. The FAR module is introduced to compensate for fine-grained high-frequency components affected by the wavelet transform. We apply global average pooling (GAP) followed by a fully connected (FC) layer to the enhanced output to generate a 7-dimensional global representation, denoted as $Y_{global}$. The SLFA module focuses on modeling fine-grained expression variations in key facial regions. Based on the facial keypoints, we apply an adaptive feature unit partitioning (AFUP) strategy to divide the face into four local units. Each region is resized to 128×14×14 to maintain alignment and consistent scale. These regions are processed by the feature enhancement module (FEM), which uses wavelet-based modeling to represent high-frequency textures and low-frequency structures. Following enhancement, the globally pooled and channel-concatenated regional features form the structured local feature $Y_{joint}$. The ExGR module is intended to enhance responsiveness to expression variations in highly activated facial regions. Specifically, the ExGR module extends outward from each facial keypoint (the eyes, nose, and mouth) to generate a fixed-size region of 128×7×7 centered on the corresponding keypoint. In this manner, the face is partitioned into five regions: $L_{1}$, $L_{2}$, $L_{3}$, $L_{4}$, and $L_{5}$, which correspond to the left eye, right eye, nose tip, and the left and right corners of the mouth, respectively. Each region is then processed by the FEM and then globally pooled through the FC layer to generate refined expression features. We fuse the outputs from all three modules to produce a unified feature representation for facial expression recognition. The three modules work collaboratively to model the separation of high-frequency and low-frequency features by leveraging the time–frequency locality of the wavelet transform, thereby enhancing fine-grained facial expression representation under frequency domain guidance.

### 3.1. Scale-Aware Expansion Module

In multi-scale feature extraction for facial expression recognition, traditional approaches generally depend on large convolutional kernels or deep network stacking to expand the receptive field. However, these approaches tend to generate excessive parameters and have difficulty accurately capturing frequency-structured characteristics. In contrast, wavelet transform excels in spatial frequency localization, making it effective for extracting both local textures and global structures in facial features. Compared to standard convolution, wavelet convolution [27] provides a more structured decomposition of high- and low-frequency components, leading to finer-grained feature representations while maintaining a large receptive field with reduced parameter complexity. Therefore, we propose the scale-aware expansion (SAE) module to enhance multi-scale feature modeling by integrating dilated convolutions and wavelet convolution (WTConv) [27]. The SAE module utilizes dilated convolution branches with varying dilation rates, enabling the network to efficiently capture information across diverse spatial scales, thereby enhancing its adaptability and recognition of multi-scale features in images. Wavelet convolution processes low-frequency and high-frequency information separately, balancing global structure and local details. By integrating these approaches, the network’s capacity to extract and leverage fine-grained features across multiple scales is significantly enhanced, thereby facilitating a more robust and effective collaborative representation of multi-scale information.

**Figure 2:**
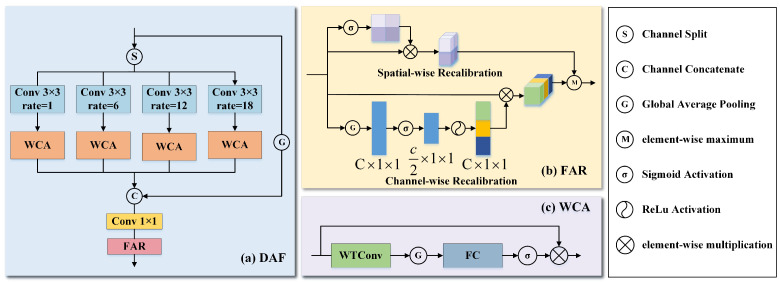
Overall architecture of the scale-aware expansion (SAE) module. (**a**) The dilated fusion (DAF) module; (**b**) the feature recalibration (FAR) module; (**c**) the wavelet transform attention (WCA).

As shown in Figure 2, the SAE module primarily consists of the dilated fusion (DAF) module and the feature recalibration (FAR) module. As depicted in Figure 2a, the DAF module extracts multi-scale features through four dilated convolution branches with different dilation rates, along with a global average pooling (GAP) branch. Each dilated branch is embedded with a wavelet transform attention (WCA) responsible for decomposing the input features into low- and high-frequency structures to improve the efficiency of multi-scale feature extraction and utilization.

**Figure 3:**
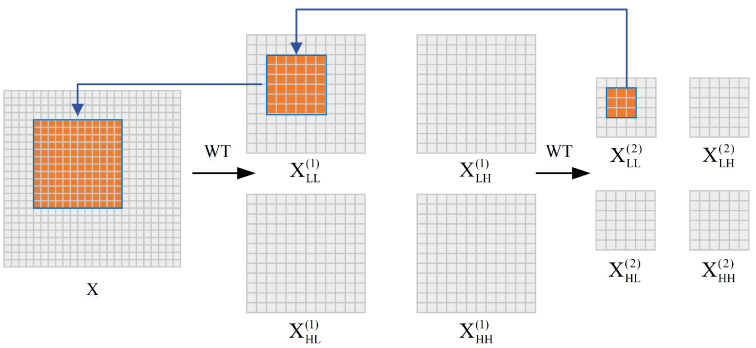
The processing procedure of wavelet transform. The input image *X* is first decomposed into one low-frequency component $X_{LL}^{\left(1\right)}$ and three directional high-frequency components: $X_{LH}^{\left(1\right)}$ (horizontal), $X_{HL}^{\left(1\right)}$ (vertical), and $X_{HH}^{\left(1\right)}$ (diagonal). The low-frequency component $X_{LL}^{\left(1\right)}$ is then further decomposed to obtain an even lower-frequency component $X_{LL}^{\left(2\right)}$, along with its corresponding high-frequency components. This hierarchical decomposition separates features across different frequencies and scales. Operating a 3×3 convolution on $X_{LL}^{\left(2\right)}$ yields a 9-parameter convolution. It demonstrates sensitivity to the lower frequencies present across a 12×12 receptive field in the primary input *X*.

In the DAF module, we divide the features extracted $F\in R^{C\times H\times W}$ from CNN equally along the channel axis into four subsets, which are then processed separately as distinct branches. Each subset is represented as $F_{i}\in R^{C_{i}\times H\times W}$, and $C_{i}=\frac{C}{4}$. C, H, and W represent channels, height, and width, respectively. The dilation rate for each branch is set as $d_{i}$. The first branch uses a 3 × 3 convolution, which maintains the spatial scale and directly extracts features. The second branch uses a 3 × 3 convolution with a dilation rate of 6 to enhance the perception of local expression features. To capture a wider range of contextual information, the third and fourth branches utilize 3 × 3 convolutions with dilation rates of 12 and 18, respectively. As shown in Figure 2c, within the wavelet transform attention (WCA), we introduce the wavelet convolution (WTConv) [27] to decompose feature maps into high-/low-frequency components, thereby obtaining deeper multi-scale features. Figure 3 shows the processing procedure of the wavelet transform. The transformation process of WTConv is as follows:

(1) Wavelet decomposition: The input image is decomposed using Haar wavelets into one low-frequency component, $W_{LL}$, along with three directional high-frequency components: $W_{LH}$, $W_{HL}$, and $W_{HH}$. $W_{LH}$, $W_{HL}$ and $W_{HH}$ correspond to the horizontal, vertical, and diagonal high-frequency components. The process is defined as follows:
  (1) $$WT\left(X\right)=\left[W_{LL},W_{LH},W_{HL},W_{HH}\right]$$
  This decomposition enables the network to capture structural and edge information separately, improving its modeling of subtle facial variations such as wrinkles and mouth corner movements.
(2) Multi-Frequency Convolution: At each layer, the low-/high-frequency parts of the input feature map are convolved with the current convolution kernel $W^{\left(i\right)}$, resulting in a low-frequency output $Y_{LL}^{\left(i\right)}$ and a high-frequency convolution output $Y_{H}^{\left(i\right)}$.
  (2) $$\begin{array}{c} \left[Y_{LL}^{\left(i\right)},Y_{H}^{\left(i\right)}\right]=\mathrm{Conv}\left(W^{\left(i\right)},\left[X_{LL}^{\left(i\right)},X_{LH}^{\left(i\right)},X_{HL}^{\left(i\right)},X_{HH}^{\left(i\right)}\right]\right) \end{array}$$
  By separating convolutions over high- and low-frequency components, the model is able to independently model structural and detailed features, enhancing its perception and discrimination of information across different frequency bands.
(3) Inverse Wavelet Transform (IWT): IWT processes the recombined convolved low- and high-frequency components to reconstruct them into a new feature map, thereby recovering the original spatial information. The process retains the original spatial structure and fuses multi-frequency responses to produce a more informative representation.
  (3) $$\begin{array}{c} X=\mathrm{IWT}(Y_{LL}^{\left(i\right)},Y_{H}^{\left(i\right)}) \end{array}$$
  Finally, after processing the four units with a global average pooling layer, we concatenate them along the channel dimension and apply an FC layer for dimensionality reduction to obtain the local features.

The GAP layer is applied initially, followed by an FC layer to establish the correlation between channels. This correlation is then normalized using the sigmoid activation function. The result is multiplied element-wise with the original features to generate the output. We concatenate the feature information $E_{i}$ from each dilated convolution branch along the channel dimension and then combine it with the features extracted from the GAP branch. The dimensionality is then reduced using a 1 × 1 convolution. The definition can be expressed as follows:

(4) $$\begin{array}{cc} M(X) & =\sigma \left(\mathrm{FC}\left(\mathrm{AvgPool}\left(\mathrm{WTConv}(X)\right)\right)\right)⊗X \end{array}$$

(5) $$\begin{array}{cc} Y & ={\mathrm{Conv}}_{1\times 1}\left(C(E_{1},\ldots ,E_{4},G)\right) \end{array}$$

where ⊗ indicates element-wise multiplication, σ stands for the sigmoid activation function, and C denotes channel concatenation, concatenating features along the channel dimension.

To mitigate the potential loss of fine-grained information in high-frequency feature modeling caused by the wavelet transform, we introduce the feature recalibration (FAR) module. Figure 2b illustrates that the FAR module serves as a complementary enhancement to our wavelet transform attention (WCA). The FAR module is inspired by the convolutional block attention module (CBAM) [63]. As shown in Figure 2c, this module combines channel-wise and spatial-wise recalibration mechanisms. To perform channel-wise recalibration, GAP is employed to produce a feature vector of size C×1×1, which is then passed through an FC layer to reduce its dimensionality to $\frac{C}{2}$. Following the ReLU activation, a second fully connected layer is employed to restore the feature dimension to the original size *C*. Finally, we perform an element-wise multiplication between the recalibrated feature vector and the original feature map to achieve channel-wise calibration. The recalibrated vector is then multiplied element-wise with the original feature map to enhance key channel responses. This effectively compensates for the local weakening of high-frequency information extraction caused by the wavelet transform. To compensate for the limitation of the wavelet transform in modeling spatial locality, we adopt spatial-wise recalibration. Specifically, a 1 × 1 convolutional operation is performed to formulate a spatial attention map. The feature map is then normalized using the sigmoid function and multiplied element-wise with the input feature map to perform spatial recalibration. The output is given by:

(6) $$Y=\max(Y_{\mathrm{Channel}},Y_{\mathrm{Spatial}})$$

Subsequently, global average pooling (GAP) is applied, followed by a fully connected (FC) layer to obtain the global representation $Y_{global}$.

The scale-aware expansion (SAE) module leverages the spatial frequency locality of wavelet transforms to achieve high- and low-frequency decomposition, enabling more effective multi-scale modeling. Guided by frequency domain information, this approach improves the capability to capture detailed facial expressions at multiple scales. In Section 4.4.2, we perform an ablation analysis to evaluate the effect of different dilation rate settings.

### 3.2. Structured Local Feature Aggregation Module

In the task of FER, accurately extracting local facial features is important. To adapt to the changing local feature distribution, we propose a structured local feature aggregation (SLFA) module for facial expression recognition. Local features are located through an adaptive feature unit partitioning strategy. The modeling of multi-scale structural details is enhanced using the feature enhancement module (FEM).

We apply standard convolution to capture basic spatial features. Within local subgraphs, we perform (WTConv) [27] for multi-frequency decomposition, extracting main structures and edge responses. We use the efficient local attention (ELA) [64] to adaptively weight different frequency components. The adaptive feature unit partitioning (AFUP) strategy is based on facial keypoint information. To achieve adaptive partitioning, the coordinates of facial keypoints are used to dynamically compute the partition boundaries for each image. This strategy allows the system to adapt to variations in facial geometry, pose, and expression. Compared with fixed-grid methods, this keypoint-based partitioning offers more semantic alignment and structural flexibility, ensuring that each region closely corresponds to meaningful facial components (e.g., eyes, mouth).

**Figure 4:**
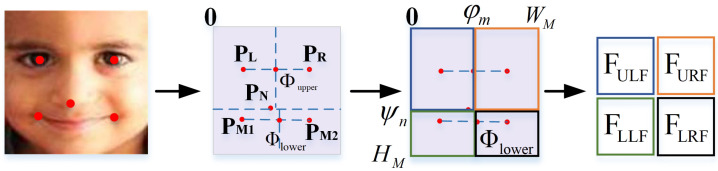
The adaptive feature unit partitioning (AFUP) strategy. Based on facial keypoints, the face is divided into four regions, namely the left-upper region, right-upper region, left-lower region, and right-lower region, denoted as $F_{ULF}$, $F_{URF}$, $F_{LLF}$, and $F_{LRF}$, respectively.

As shown in Figure 4, first, we use the RetinaFace [62] detector to extract five facial keypoints: $P=\{P_{L},P_{R},P_{N},P_{M1},P_{M2}\}$, where $P_{L}=\left(\varphi_{L},\psi_{L}\right)$, $P_{R}=\left(\varphi_{R},\psi_{R}\right)$, $P_{N}=\left(\varphi_{N},\psi_{N}\right)$, $P_{M1}=\left(\varphi_{M1},\psi_{M1}\right)$, and $P_{M2}=\left(\varphi_{M2},\psi_{M2}\right)$ denote the centers of the left and right eyes, the tip of the nose, and the corners of the mouth, respectively. Here, φ and ψ correspond to the horizontal and vertical coordinate values. To reasonably divide the facial feature units, we introduce a horizontal boundary line and two vertical boundary lines to define the boundaries of the upper, lower, left, and right feature units. The horizontal boundary line is based on the coordinate $P_{N}=\left(\varphi_{N},\psi_{N}\right)$ of the nose tip, which serves as the boundary between the upper and lower feature units. The upper vertical boundary line is based on the midpoint of the coordinates of the left and right eyes, denoted as $\Phi_{\mathrm{upper}}=\left(\varphi_{m},\psi_{m}\right)$, and the lower vertical boundary line is based on the midpoint of the coordinates of the left and right mouth corners, denoted as $\Phi_{\mathrm{lower}}=\left(\varphi_{n},\psi_{n}\right)$. The definitions of $\Phi_{\mathrm{upper}}$ and $\Phi_{\mathrm{lower}}$ are as follows:

(7) $$\begin{array}{cc} \varphi_{m} & =\frac{\varphi_{L}+\varphi_{R}}{2} \end{array}$$

(8) $$\begin{array}{cc} \psi_{m} & =\frac{\psi_{L}+\psi_{R}}{2} \end{array}$$

(9) $$\begin{array}{cc} \varphi_{n} & =\frac{\varphi_{M1}+\varphi_{M2}}{2} \end{array}$$

(10) $$\begin{array}{cc} \psi_{n} & =\frac{\psi_{M1}+\psi_{M2}}{2} \end{array}$$

Based on these facial keypoints, the facial feature map X is divided as follows:

(11) $$F_{ULF}=S[0:\varphi_{m},\;0:\psi_{n}]$$

(12) $$F_{URF}=S[\varphi_{m}:W_{M},\;0:\psi_{n}]$$

(13) $$F_{LLF}=S[0:\varphi_{m},\;\psi_{n}:H_{M}]$$

(14) $$F_{LRF}=S[\varphi_{m}:W_{M},\;\psi_{n}:H_{M}]$$

where $F_{ULF}$, $F_{URF}$, $F_{LLF}$, and $F_{LRF}$ correspond to the left-upper, right-upper, left-lower, and right-lower feature units. *S* denotes the partitioning of the feature map *X* into regions, and the number 0 indicates extraction starting from the top-left corner of the image. $H_{M}$ and $W_{M}$ correspond to the maximum coordinates of the feature map in the vertical and horizontal directions. To ensure consistency in feature extraction scales, if the width or height of a local feature region is smaller than the predefined minimum size $S_{\mathrm{target}}$, we calibrate and zero-pad the local units. According to the suggestion of AMP-Net [54], each feature unit is uniformly set to the scale of 14 × 14.

**Figure 5:**
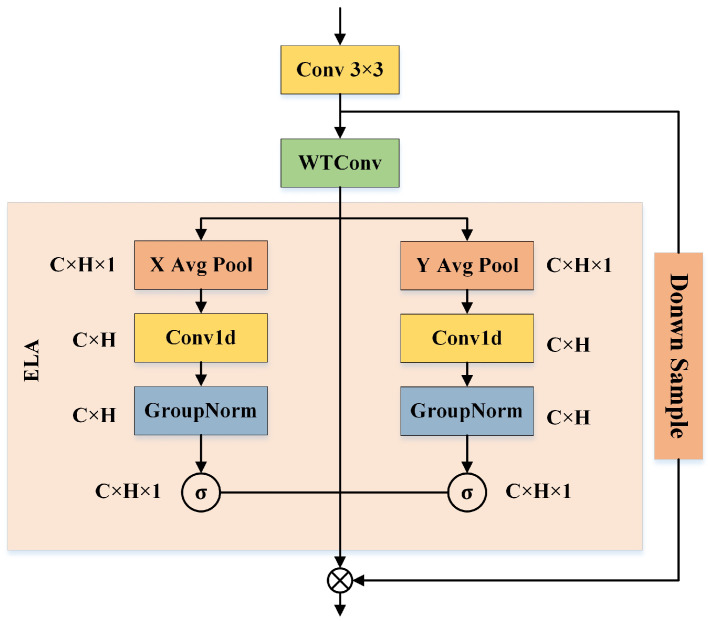
The overall architecture of the feature enhancement module (FEM). ELA denotes the efficient local attention.

We further enhance the local feature representation of each feature unit using the feature enhancement module (FEM). The goal of the FEM is to enrich local structural cues in each region by integrating spatial, frequency, and directional information. Specifically, standard convolution captures basic spatial context, WTConv [27] extracts hierarchical frequency components, and ELA [64] emphasizes directional attention patterns. This design enhances the model’s sensitivity to subtle facial variations, thereby improving both the accuracy and robustness of facial expression recognition. As shown in Figure 5, each local unit is processed in sequence using a 3×3 convolution, wavelet convolution (WTConv) [27], and the efficient local attention (ELA) [64]. The enhanced feature representation is then produced through element-wise fusion. WTConv [27] performs frequency domain decomposition on local features, separating them into low-frequency and high-frequency components. Unlike in global contexts, WTConv [27] in the FEM focuses on local sub-regions, emphasizing the preservation and separation of fine-grained hierarchical information. We then introduce the ELA to perform directional modeling on each frequency component. The main steps of ELA [64] include coordinate information embedding, position attention generation, and the final attention map. For the given feature map X, ELA [64] first uses strip pooling to extract the global features along the horizontal axis $u_{p}$ and vertical axis $v_{q}$:

(15) $$\begin{array}{c} u_{p}=\frac{1}{H}\sum_{i=0}^{H-1}F\left(p,i\right) \end{array}$$

(16) $$\begin{array}{c} v_{q}=\frac{1}{W}\sum_{i=0}^{W-1}F\left(j,q\right) \end{array}$$

where F(p,i) represents the horizontal pixel values and F(j,q) represents the vertical pixel values. To enhance feature interaction, the extracted features are processed through a 1 × 1 convolution, and the group normalization layer is used to improve generalization ability. Finally, by merging the horizontal attention weight $a_{p}$ with the vertical attention weight $b_{p}$, the enhanced feature map is defined as:

(17) $$\begin{array}{c} F_{ab}=F\times a_{p}\times b_{q} \end{array}$$

Finally, after processing the four units with a GAP layer, we concatenate them along the channel dimension and apply an FC layer for dimensionality reduction, leading to the local features. By combining adaptive region partitioning guided by facial keypoints with localized feature enhancement via the FEM, the proposed SLFA module aims to improve structural consistency and semantic expressiveness of local features, which contribute to more accurate and robust facial expression recognition.

### 3.3. Expression-Guided Region Refinement Module

To better model high-response facial regions, we propose the expression-guided region refinement (ExGR) module. Compared with the SLFA module, ExGR extends facial landmarks into fixed regions, with a focus on areas exhibiting the most pronounced expression variations. The areas around the eyes and mouth corners are particularly informative and offer stronger discriminative information for expression recognition.

In response to this property, the ExGR module divides the face into five regions: $L_{1}$, $L_{2}$, $L_{3}$, $L_{4}$, and $L_{5}$, which correspond to the left eye, the right eye, the nose tip, and the left and right corners of the mouth. Each region is centered on its corresponding facial keypoint. Specifically, for each facial landmark, the ExGR module constructs a fixed-size M × M region centered at the corresponding keypoint. This fixed-scale region strategy is designed to cover the most expression-sensitive facial areas and to enhance the representation of critical local features. For each region, features are further refined using the feature enhancement module (FEM) to capture both high-frequency textures and low-frequency structures. The five regional features are fused through channel concatenation to form a unified representation of key expression areas. The fused feature is then processed with the GAP layer for spatial reduction, followed by an FC layer for compression and projection, obtaining the key region feature $F_{key}$.

The ExGR module complements SLFA by focusing on expression dynamics in key regions, improving the modeling of both local structures and fine-grained expression variations. In Section 4.4.4, we analyze the impact of the region size M through ablation studies. In Section 4.5, we provide visualizations of the five expression regions processed by the ExGR module to further illustrate the areas of attention learned by the model.

### 3.4. Fusion Strategy and Loss Function

We combine the feature outputs from the SLFA and ExGR modules through concatenation along the channel dimension during the early phase of feature fusion. The joint feature vector $Y_{joint}$ is obtained by applying a fully connected layer to compress the feature dimensionality. During the decision stage, the global discriminative feature vector $Y_{global}$ output by the SAE module is fused with the joint feature vector $Y_{joint}$ through a weighted strategy to further enhance classification accuracy. We utilize an adjustable hybrid loss strategy to effectively integrate global and local features. This strategy is defined as follows:

(18) $$Y=\alpha Y_{global}+\left(1-\alpha \right)Y_{joint}$$

where α∈[0,1] is the fusion factor that controls the contribution of global information and joint features. We use the cross-entropy loss to compute both Yglobal and $Y_{joint}$, formulated as follows:

(19) $$Y_{M}=-\frac{1}{N}\sum_{i=1}^{N}\sum_{j=1}^{C}t_{j}^{\left(i\right)}\log{\hat{t}}_{j}^{\left(i\right)}$$

where N denotes the number of samples, $t_{j}^{\left(i\right)}$ represents the ground-truth label of the i-th sample for class j, and ${\hat{t}}_{j}^{\left(i\right)}$ is the predicted probability for class j of the i-th sample.

## 4. Experimental Verification

### 4.1. Datasets

To evaluate the effectiveness of the WGGLFA, we perform extensive experiments on three real-world facial expression datasets: RAF-DB [13], FERPlus [65], and FED-RO [16].

RAF-DB [13] is a real-world dataset for facial expression recognition, containing 7 basic expressions and 11 compound expressions. The experimental data consisted of 7 basic expressions, with 12,271 images used to train the model and 3068 images allocated for testing.

FERPlus [65] is an expanded and re-annotated version of the FER2013 [66] dataset. The dataset consists of 28,709 training images, 3589 validation images, and 3589 test images. In the experiments, we used seven basic expressions along with contempt as the training data.

FED-RO [16] is a facial occlusion dataset obtained from Bing and Google search and excluding images overlapping with RAF-DB [13] and AffectNet [14]. The images exhibit diverse occlusion patterns in color, shape, position, and degree of coverage, comprising 400 samples across seven expression categories.

### 4.2. Experiment Details

1. Data Preparation: In all our experiments, we utilized aligned image samples provided by the official dataset. Each input image was resized to 224×224, and five predefined facial keypoints were detected using RetinaFace [62].
2. Training: ResNet-34 [45] was employed as the backbone network, with its parameters initialized using ImageNet pre-trained weights. Stochastic gradient descent (SGD) was employed as the optimization algorithm, initialized with a learning rate of 0.01, which decayed every 20 epochs. The model was trained for 100 epochs, applying early stopping when appropriate to avoid overfitting. The model was trained using a batch size of 64, a momentum value of 0.9, and a weight decay of 0.0001. Our WGGLFA contains 53.74 million parameters and 1.42 G FLOPs. We implemented the model using PyTorch 1.11.0, and all experiments were performed using an NVIDIA A100 GPU equipped with 40 GB of memory.

### 4.3. Comparison with State-of-the-Art Methods

In this section, we compare our best results with several state-of-the-art methods on the RAF-DB, FERPlus, and FED-RO datasets. As shown in Table 1, WGGLFA achieves facial expression recognition accuracy of 90.32% and 91.24% on RAF-DB and FERPlus, respectively. Compared to the recent state-of-the-art method LCFC [67], our method achieves 1.09% and 1.60% higher accuracy on RAF-DB and FERPlus, respectively. LCFC lacks explicit modeling of key expression regions, which limits its ability to capture fine-grained facial changes. In contrast, our method integrates global semantic information with local details to effectively model the multi-scale structural characteristics inherent in facial expressions. This capability stems from the use of the wavelet transform to perform high–low frequency feature decomposition, which enhances the representation of fine-grained facial details under frequency domain guidance. On the RAF-DB dataset, WGGLFA outperforms Twinned-Att [68], MM-Net [46], AMP-Net [54], and DENet [69] by 3.4%, 0.55%, 1.07%, and 2.97%, respectively. On FERPlus, it achieves higher accuracy than MM-Net [46] (1.9%), AMP-Net [54] (5.8%), and DENet [69] (1.87%). These comparisons demonstrate the effectiveness of our global–local fusion strategy across different datasets. In the FERPlus dataset, Twinned-Att [68] did not include the contempt category during training; therefore, its results are excluded from the comparison to ensure fairness. To better understand the above performance improvements, we further analyze the architectural and design differences between our method and the compared approaches. MM-Net [46] suffers from information loss during multi-scale feature fusion. Both AMP-Net [54] and Twinned-Att [68] also use global features and focus on small local regions. In addition, Twinned-Att [68] relies more on predefined facial keypoints, while WGGLFA effectively extracts multi-scale features while maintaining a good balance between performance and model size. In terms of computational complexity, WGGLFA shows a clear advantage.

As shown in Table 1, our WGGLFA network achieves state-of-the-art performance with only 1.42 GFLOPs, which is significantly lower than that of MM-Net (4.70 G) [46], Twinned-Att (4.96 G) [68], and MA-Net (3.65 G) [47]. Although AMP-Net [54] has slightly fewer FLOPs (1.69 G), it comes with a much larger parameter size of 105.67 M. In contrast, WGGLFA reduces the number of parameters by 51.93 M. WGGLFA effectively improves facial expression recognition performance while optimizing computational efficiency and model capacity.

Table 2 presents the classification accuracy of different models across seven emotion categories evaluated on the RAF-DB dataset. WGGLFA achieves the highest accuracy in four categories: surprise (0.89), fear (0.77), happy (0.97), and neutral (0.92). Table 3 provides a comparative evaluation of classification accuracy across eight emotion categories on the FERPlus dataset. Our method also achieves the highest accuracies for the three facial expressions (happy, angry, and surprise) among these methods, which are 0.96, 0.92, and 0.90, respectively. We further compared the classification accuracy of the WGGLFA network with other models across seven emotion categories on the FED-RO dataset. As shown in Table 4, the WGGLFA network performs well in recognizing fear and anger (0.77 and 0.84), while its performance on disgust is comparatively limited.

**Figure 6:**
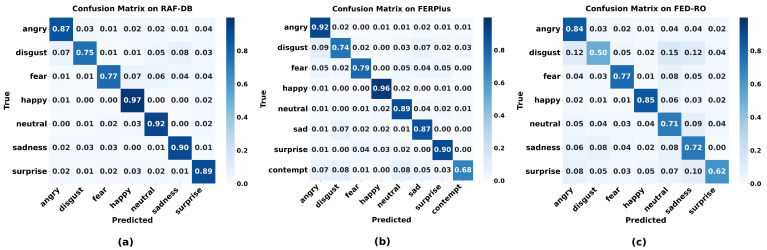
Confusion matrices of facial expression recognition performance on the RAF-DB, FERPlus, and FED-RO datasets. (**a**) Confusion matrix on RAF-DB. (**b**) Confusion matrix on FERPlus. (**c**) Confusion matrix on FED-RO.

We further evaluate the classification effectiveness of WGGLFA within core facial expression categories using confusion matrix analysis. As shown in Figure 6a, WGGLFA achieves an accuracy of 97% in recognizing happy on RAF-DB. However, its performance is lower when recognizing negative affective states, including disgust and fear. For instance, 7% of angry samples and 8% of sad samples are misclassified as disgust. This may be due to shared facial features among these expressions, such as downturned mouth corners and furrowed brows. Figure 6b presents the confusion matrix across eight expression categories on FERPlus. WGGLFA yields high recognition accuracies for happy (0.96), angry (0.92), and surprise (0.90), whereas the recognition accuracy for contempt is relatively low (0.68). Finally, we evaluate the generalization ability of the model by testing it on FED-RO using the model trained on RAF-DB. Table 1 shows that the proposed method achieves an accuracy of 71.90%, which is higher than the other compared techniques. Figure 6c presents the confusion matrix on FED-RO, which shows that WGGLFA achieves relatively high recognition rates for happy (0.85) and angry (0.84) expressions. In contrast, the recognition accuracy for disgust is lower, reaching 0.50, with frequent misclassifications as neutral or sadness. This may be due to the subtle and localized nature of disgust-related facial movements, such as slight wrinkling of the nose and gentle raising of the upper lip, which may resemble the expressions of neutral or mildly negative emotional states, thus leading to confusion. These experimental results indicate that WGGLFA exhibits strong robustness in handling occlusion challenges.

### 4.4. Ablation Study

#### 4.4.1. Effectiveness of the Proposed Modules

To validate the modules proposed in WGGLFA, we conducted an ablation experiment to explore the impact of the SAE, SLFA, and ExGR modules on the RAF-DB, FER-Plus, and FED-RO datasets. Table 5 provides a comprehensive summary of the results. The baseline model is based on a standard ResNet-34 architecture without incorporating the SAE, SLFA, or ExGR modules, serving as a reference for evaluating the effectiveness of each component. Incorporating the SAE module leads to recognition accuracy improvements of 1.88%, 1.52%, and 5.54% on RAF-DB, FERPlus, and FED-RO, respectively. The observed performance improvement is attributed to the use of the wavelet transform, which decomposes input features into high-frequency and low-frequency components and enables effective multi-scale context modeling. The SLFA module further improves performance by 1.14%, 1.29%, and 4.60% on the three datasets by introducing dynamic region partitioning and enhancing local feature aggregation. Wavelet transform and local attention are applied within each region to jointly model structural and edge features across multiple frequency components, thereby enhancing the discriminability and completeness of local representations. The ExGR module enhances accuracy to 85.91% on RAF-DB, 86.02% on FERPlus, and 63.44% on FED-RO by improving local feature extraction through fixed-region modeling.

We conducted an additional set of ablation experiments to investigate the collaborative contribution of the three modules. As presented in Table 5, removing the ExGR module reduces accuracy to 88.02% on RAF-DB, 88.69% on FERPlus, and 68.60% on FED-RO, confirming its complementary role in modeling key region features. The fixed-region strategy consistently targets high-response areas, thereby enhancing the representation of critical facial regions. The removal of the SLFA module leads to accuracy reductions of 1.55%, 2.20%, and 2.40% on the three datasets, respectively, demonstrating the effectiveness of the AFUP strategy in aggregating local features and extracting fine-grained facial details. Removing the SAE module causes performance drops of 2.65%, 2.61%, and 2.65% on the three datasets, respectively, highlighting its role in capturing multi-scale expression structures and enhancing scale-aware representations. This result further suggests that SLFA and EXGR provide complementary capabilities in local feature modeling, jointly enhancing the representation of fine-grained details at multiple spatial scales. The full model (the combined effect of SAE, SLFA, and ExGR modules) achieved the highest recognition accuracy of 89.15%, 89.44%, and 69.90% on RAF-DB, FERPlus, and FED-RO, respectively, without pretraining.

The results validate the complementary roles of the three modules in multi-scale context modeling, structured local feature extraction, and key region enhancement, leading to improved accuracy and generalization in facial expression recognition.

#### 4.4.2. The Impact of the Dilation Rate d

To ensure the credibility of our research, we conducted an independent experiment to explore the potential impact of varying receptive field sizes on feature extraction by adjusting the dilation rate *d* in different branches of the dilated fusion (DAF) module. We set $d_{1}$ in the first branch to 1 (the smallest receptive field) with no dilation, used for direct feature extraction. Then, we gradually increased the sizes of $d_{2}$, $d_{3}$, and $d_{4}$. The experimental results are illustrated in Table 6. The model achieved its highest classification accuracy with the dilation rate combination of (1, 6, 12, 18), reaching 89.15% on RAF-DB, 89.44% on FERPlus, and 69.90% on FED-RO. The results indicate that as the dilation rate increases, the accuracy of the model gradually improves. This result indicates that appropriately increasing the dilation rate can effectively expand the receptive field, thereby enabling it to capture broader global features in the image.

#### 4.4.3. The Impact of the Fusion Factor α

**Figure 7:**
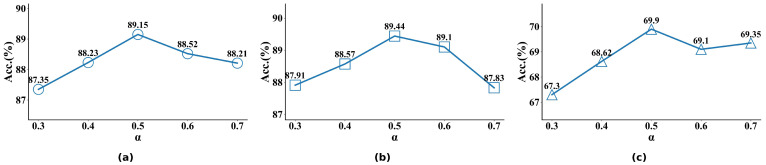
Impact of the fusion factor α on the RAF-DB, FERPlus, and FED-RO datasets. (**a**) Results on RAF-DB. (**b**) Results on FERPlus. (**c**) Results on FED-RO.

The fusion factor α is used to control the ratio of global features to joint features in the fusion process. Specifically, the size of α determines the weight of each feature in the final model output. The relationship between the fusion factor α and model performance is presented in Figure 7. The experimental results show that when the fusion factor α is set to 0.5, WGGLFA achieves its best performance on all three datasets, reaching classification accuracies of 89.15%, 89.44%, and 69.9% on RAF-DB, FERPlus, and FED-RO, respectively. Under this configuration, the model fully exploits the complementary strengths of global and local features, resulting in a well-balanced fusion. We observe that when α is excessively high or low, it can cause one feature type to be overemphasized and another important feature to be neglected, resulting in degraded model performance.

#### 4.4.4. The Impact of the Region Size M

We conducted an ablation experiment by adjusting the size of M in ExGR to analyze its impact on the model’s performance. The experimental results are presented in Table 7. When (M = 7), the model achieves the highest accuracy of 89.15% on RAF-DB, 89.44% on FERPlus, and 69.90% on FED-RO, suggesting that this configuration provides an optimal trade-off between spatial resolution and semantic coverage. In contrast, smaller region sizes (e.g., (M = 5)) lead to excessively narrow partitions, potentially missing critical facial expression cues. Conversely, larger values (e.g., (M = 9) or (M = 10)) produce overly coarse divisions, which may dilute fine-grained expression features. These results indicate that a moderate region size facilitates the effective capture of local facial expression variations. Accordingly, M = 7 is chosen as the optimal setting in our framework.

### 4.5. Visualization Analysis

To more clearly illustrate the effectiveness of our WGGLFA network, we used the GradCAM [70] method to visualize image heatmaps. Specifically, we resized the attention map for visualization to align with the dimensions of the input image. Subsequently, gradient information was propagated backward to the feature outputs of the convolutional network to produce heatmaps that emphasize regions relevant to specific classes.

**Figure 8:**
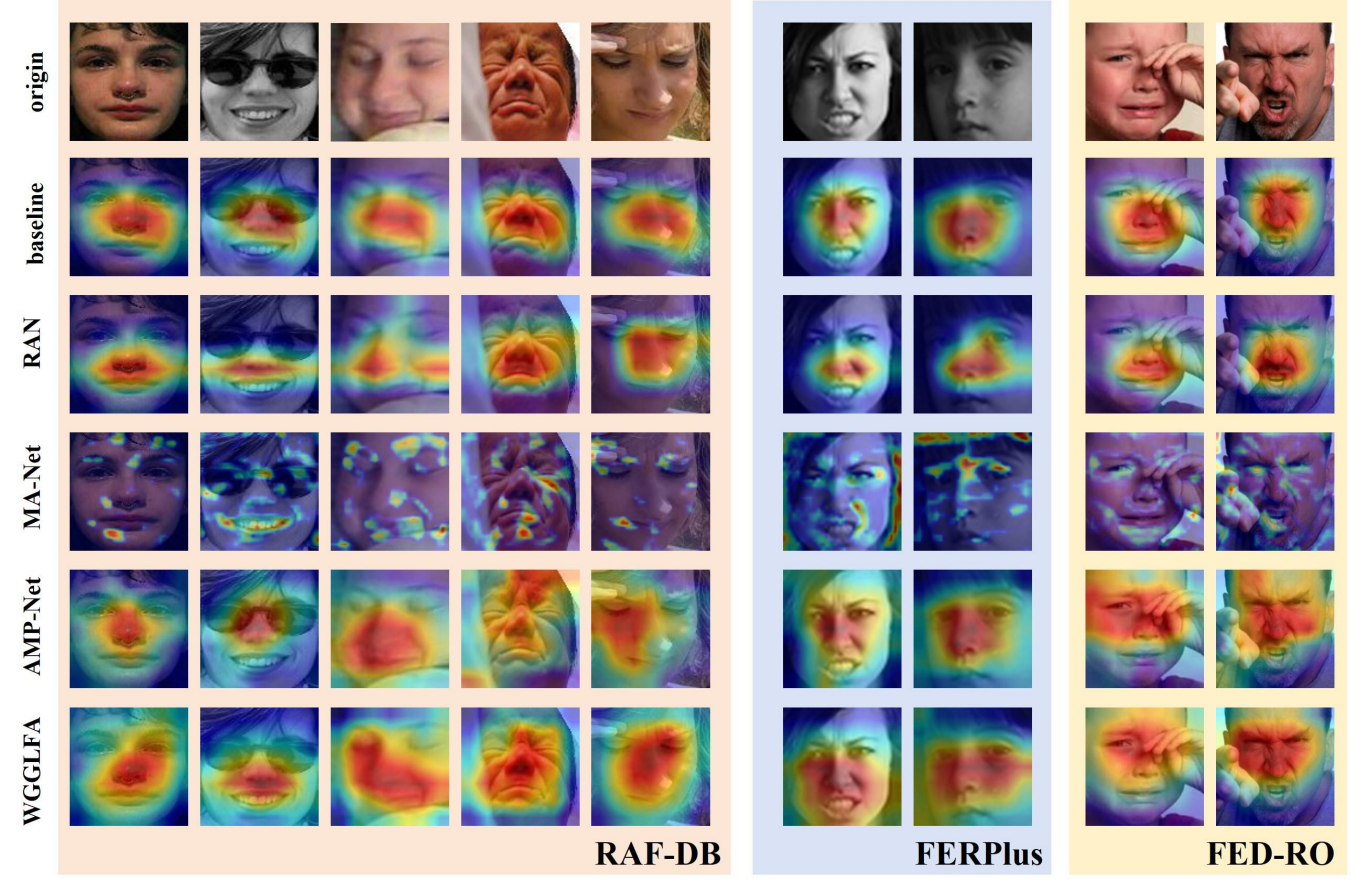
The visualization comparison of different facial expression recognition methods on sample face images.

Figure 8 presents our WGGLFA network alongside four publicly available implementations, illustrating their visualization results on three datasets. Since the source code for some methods is not publicly available, they are omitted from the figure. Each column corresponds to a single input sample: the first row shows the original image, and each subsequent row displays the visualization produced by a different method. The baseline method is ResNet-34. For unoccluded samples, as shown in the first, fifth, and sixth columns, our WGGLFA network effectively concentrates attention on regions closely related to facial expressions, covering the entire face and particularly focusing on key areas such as the eyes, the tip of the nose, and the mouth. In contrast, the baseline method and AMP-Net [54] exhibit a more diffuse attention distribution when processing the same samples. The attention maps generated by RAN [17] and MA-Net [47] display shifts or dispersion in several cases, such as in the samples from the third, seventh, and ninth columns, with portions of attention falling on the background or irrelevant regions, which may impair the model’s ability to perceive expressions. In the case of occluded samples, such as the second-column sample, the WGGLFA network is still able to focus on emotion-related regions despite the eye area being obstructed.

**Figure 9:**
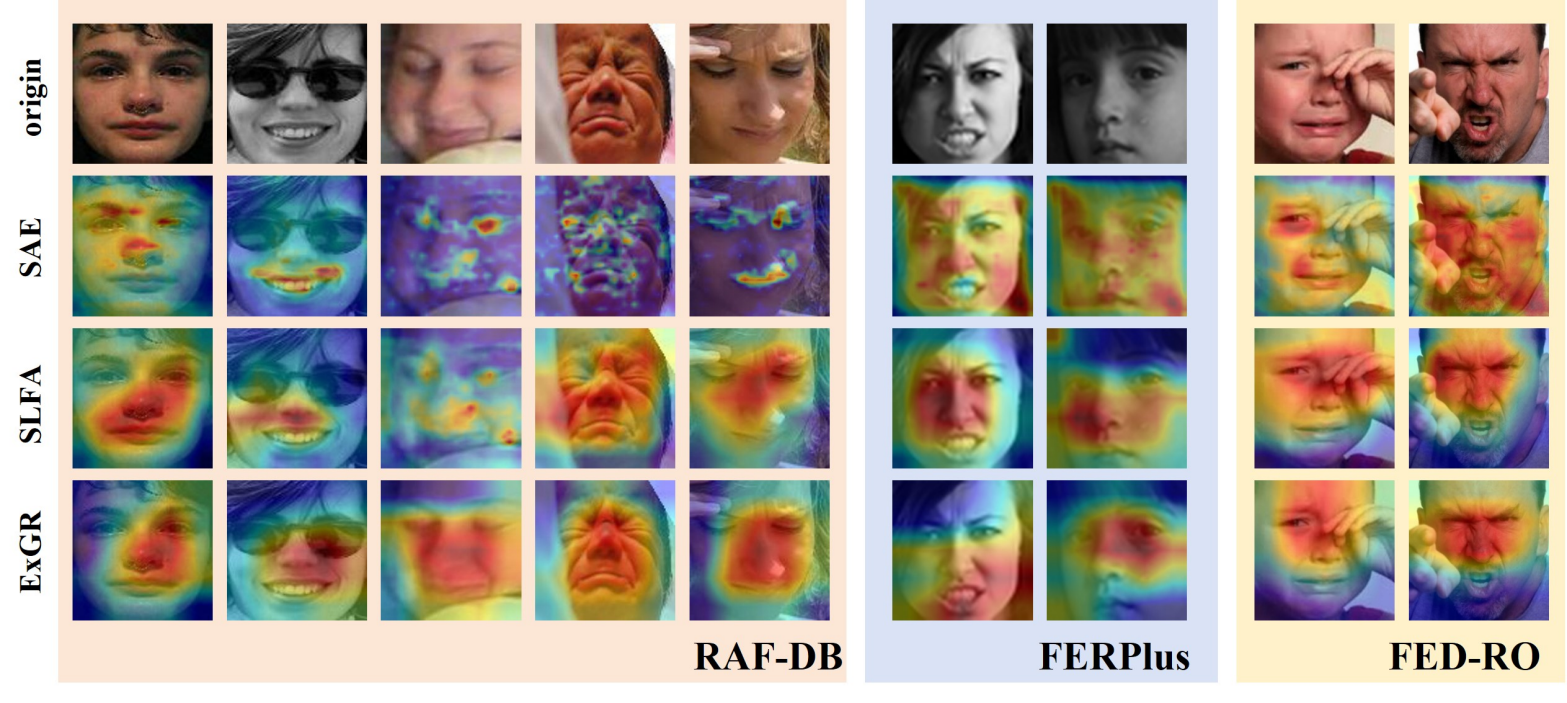
The visualization comparison of the various modules of our WGGLFA network on sample facial images.

**Figure 10:**
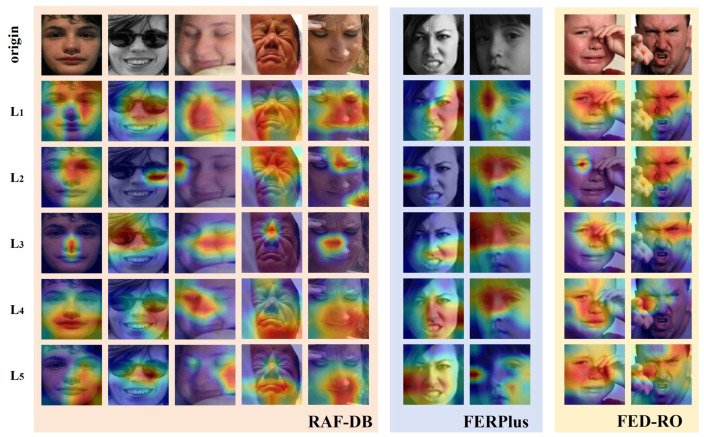
The visualization of different regions in the ExGR module on example face images. $L_{1}$–$L_{5}$ represent predefined semantic regions centered on key facial keypoints, where $L_{1}$ and $L_{2}$ correspond to the left and right eyes, $L_{3}$ to the nose tip, and $L_{4}$ and $L_{5}$ to the left and right corners of the mouth, respectively.

To further validate the effectiveness of each submodule within WGGLFA, we conducted a visualization analysis of its three core components: the SAE, SLFA, and ExGR modules. As shown in Figure 9, the SAE module not only captures fine-grained local facial details but also extracts more global expression information when processing diverse facial expressions. In the second-column sample, even under local occlusion caused by eyeglasses, the SAE module still effectively focuses on regions that are discriminative for expression recognition. This robustness arises from the spatial–frequency localization provided by the wavelet transform combined with the receptive-field expansion of dilated convolutions, which together enhance contextual feature modeling. After incorporating the SLFA module, the model in the fourth- and fifth- column samples demonstrates stronger local feature modeling capabilities, accurately concentrating on key expression areas such as the eyes and mouth. The heatmaps produced by the ExGR module illustrate how it defines fixed regions based on expression priors and then precisely attends to the most significant expression change regions. In the eighth column sample, despite hand occlusion, the ExGR module shows markedly increased attention on the eye area, indicating its enhanced ability to model critical expression features. To better observe the contribution of the ExGR module, we visualized the five regions processed by the ExGR module ($L_{1}$, $L_{2}$, $L_{3}$, $L_{4}$, and $L_{5}$). As shown in Figure 10, region $L_{3}$, corresponding to the nose area, exhibits higher activation than the other regions. In the case of hand occlusion, the $L_{2}$ activation map for the eighth-column sample still exhibits a strong response in the eye region. Based on the above analysis, WGGLFA demonstrates excellent performance in facial expression recognition, validating its broad applicability and strong potential in practical scenarios.

## 5. Conclusions

In this paper, we propose a wavelet-guided global–local feature aggregation network (WGGLFA). The network extracts multi-scale features from facial expressions, aggregates local features, and guides attention to key regions associated with facial expressions. We propose the scale-aware expansion (SAE) module, which combines wavelet transform and dilated convolution to capture multi-scale contextual features and better represent facial structure and texture. The structured local feature aggregation module adaptively partitions facial regions based on key points and leverages wavelet-based frequency features to model local structures. The expression-guided region refinement module focuses attention on high-response emotional regions, enabling joint modeling of critical facial details and expression-relevant areas. All three modules utilize wavelet transform to separate frequency components and integrate multi-scale features, enhancing fine-grained expression representation. Experimental results show that WGGLFA outperforms the current state of the art on publicly available datasets (RAF-DB, FERPlus, and FED-RO).

## 6. Limitations and Future Work

Although WGGLFA demonstrates strong representational ability in static-image FER tasks, its current architecture is restricted to the visual modality and does not incorporate auxiliary signals such as speech or text. This design focuses on evaluating the wavelet-guided global–local feature aggregation mechanism under unimodal conditions. In future work, we aim to extend the framework to multimodal emotion recognition by incorporating complementary audio and linguistic information and to evaluate its performance in more complex and interactive scenarios.

## Figures and Tables

**Table 1:** Comparison with state-of-the-art results on the RAF-DB, FERPlus, and FED-RO datasets. The magnitudes for FLOPs and parameters are measured in GIGA (10^9^) and MEGA (10^6^), respectively.

Methods	Accuracy (%)			Params (M)	FLOPs (G)
	RAF-DB	FERPlus	FED-RO		
RAN [17]	86.90	88.55	67.98	–	–
MA-Net [47]	88.40	87.60	70.00	50.54	3.65
AMP-Net [54]	89.25	85.44	71.75	105.67	1.69
MM-Net [46]	89.77	89.34	68.75	23.11	4.70
DENet [69]	87.35	89.37	71.50	–	–
Twinned-Att [68]	86.92	–	69.82	52.85	4.96
LCFC [67]	89.23	89.60	–	22.61	–
WGGLFA (Ours)	90.32	91.24	71.90	53.74	1.42

**Table 2:** Accuracy comparison of different models on seven emotion categories on RAF-DB.

Emotion	RAN [17]	MA-Net [47]	AMP-Net [54]	MM-Net [46]	DENet [69]	Twinned-Att [68]	LCFC [67]	WGGLFA (Ours)
neutral	0.81	0.85	0.89	0.88	0.88	0.88	0.89	0.92
fear	0.76	0.76	0.65	0.75	0.66	0.73	0.68	0.77
disgust	0.68	0.67	0.65	0.73	0.54	0.86	0.72	0.75
happy	0.87	0.93	0.96	0.96	0.95	0.85	0.94	0.97
sadness	0.75	0.79	0.87	0.89	0.85	0.92	0.89	0.90
angry	0.85	0.84	0.82	0.85	0.80	0.87	0.83	0.87
surprise	0.78	0.86	0.86	0.86	0.87	0.86	0.87	0.89

**Table 3:** Accuracy comparison of different models on eight emotion categories on FERPlus.

Emotion	RAN [17]	MA-Net [47]	AMP-Net [54]	MM-Net [46]	DENet [69]	LCFC [67]	WGGLFA (Ours)
neutral	0.83	0.85	0.83	0.88	0.92	0.92	0.89
fear	0.80	0.82	0.79	0.78	0.54	0.53	0.79
disgust	0.76	0.77	0.65	0.65	0.53	0.56	0.74
happy	0.88	0.92	0.91	0.95	0.95	0.96	0.96
sadness	0.76	0.83	0.81	0.81	0.78	0.79	0.87
angry	0.87	0.90	0.88	0.91	0.89	0.86	0.92
surprise	0.82	0.86	0.84	0.89	0.92	0.93	0.90
contempt	0.50	0.51	0.68	0.62	0.38	0.31	0.68

**Table 4:** Accuracy comparison of different models on seven emotion categories on FED-RO.

Emotion	RAN [17]	MA-Net [47]	AMP-Net [54]	Twinned-Att [68]	WGGLFA (Ours)
neutral	0.68	0.70	0.70	0.72	0.71
fear	0.65	0.75	0.76	0.67	0.77
disgust	0.62	0.48	0.47	0.66	0.50
happy	0.80	0.83	0.86	0.74	0.85
sadness	0.66	0.70	0.74	0.69	0.72
angry	0.64	0.78	0.83	0.63	0.84
surprise	0.63	0.60	0.63	0.73	0.62

**Table 5:** Evaluation of each module on the RAF-DB, FERPlus, and FED-RO datasets without pre-training. SAE denotes the scale-aware expansion module, SLFA denotes the structured local feature aggregation module, and ExGR denotes the expression-guided region refinement module.

Ablation Strategy			Accuracy (%)		
SAE	SLFA	ExGR	RAF-DB	FERPlus	FED-RO
			85.00	85.20	61.20
✓			86.88	86.72	66.74
	✓		86.14	86.49	65.80
		✓	85.91	86.02	63.44
✓	✓		88.02	88.69	68.60
✓		✓	87.60	87.24	67.50
	✓	✓	86.50	86.83	67.25
✓	✓	✓	89.15	89.44	69.90

**Table 6:** Performance of dilation rate combinations $\left(d_{1},d_{2},d_{3},d_{4}\right)$ in the DAF module on RAF-DB, FERPlus, and FED-RO without pre-training.

Dilation Rates				Accuracy (%)		
$d_{1}$	$d_{2}$	$d_{3}$	$d_{4}$	**RAF-DB**	**FERPlus**	**FED-RO**
1	2	3	4	87.35	87.89	68.85
1	2	4	6	87.81	88.33	69.12
1	2	4	8	88.50	88.75	69.30
1	4	8	12	88.14	88.50	69.50
1	6	12	18	89.15	89.44	69.90

**Table 7:** Accuracy on RAF-DB, FERPlus, and FED-RO with different region sizes.

Region Size	Accuracy (%)		
	RAF-DB	FERPlus	FED-RO
5	88.83	89.00	69.45
6	88.98	89.27	69.73
7	89.15	89.44	69.90
8	89.02	89.21	69.40
9	88.90	89.15	69.30
10	88.72	89.02	69.15

## Data Availability

The datasets used in this study are sourced from publicly available repositories.
